# Supplementary material for: Complexome profiling of the Chlamydomonas psb28 mutant reveals TEF5 as an early PSII assembly factor
Source: Plant Cell. 2025 Mar 17;37(6):koaf055. doi: 10.1093/plcell/koaf055 (PMC12164590; doi:10.1093/plcell/koaf055)
Supplement: koaf055_Supplementary_Data [file koaf055_supplementary_data.zip › psb28_interactive.zip!/plots/intensity/qualityPlots/peptides/NFO2_Cre17.g727300.t1.2.html]

### Cre17.g727300.t1.2

Trivial name: NFO2  
SeqCoverage [median]: 38.40  
Mass: 41.59 kDa  
  
 

| Sequence | MissedCleavage | Mass [Da] | wtIntensity | muIntensity | Score |
| --- | --- | --- | --- | --- | --- |
| AALPMASSLIAPLK | 0 | 1381.80 | 309.71 | 0.00 | 60.52 |
| ADYEVPR | 0 | 848.40 | 2451.70 | 7321.40 | 153.15 |
| ALSTEEVEQVPK | 0 | 1328.68 | 0.00 | 2423.92 | 163.38 |
| APGLATEAQATAWKPVVQK | 1 | 1965.07 | 0.00 | 1494.54 | 50.65 |
| DFAAAALR | 0 | 833.44 | 398.73 | 1227.64 | 76.03 |
| FLGLSEPNIK | 0 | 1116.62 | 0.00 | 2899.89 | 63.53 |
| GGILYAQLFHAGR | 0 | 1401.75 | 0.00 | 703.48 | 58.27 |
| ISMSAMTR | 0 | 895.43 | 1878.70 | 860.91 | 93.37 |
| MSPGTELPNDLVAQYYEQR | 0 | 2210.03 | 0.00 | 3580.42 | 137.45 |
| TAGLNPGGFDAK | 0 | 1146.57 | 3750.61 | 8144.87 | 160.36 |
| TDKYGGSVANR | 1 | 1166.57 | 605.93 | 602.73 | 88.80 |
| VDPATIKA | 1 | 813.46 | 0.00 | 1939.60 | 86.79 |
| VGAQQLAHR | 0 | 978.54 | 0.00 | 394.78 | 113.89 |
| YGGSVANR | 0 | 822.40 | 0.00 | 665.58 | 83.61 |
|  |  |  | 9395.4 | 32259.8 |  |
